# Prenatal diagnosis of lysosomal storage disorders: our experience in 120 cases

**DOI:** 10.1186/1755-8166-7-S1-P126

**Published:** 2014-01-21

**Authors:** Mehul Mistri, Nrupesh Oza, Frenny Sheth, Jayesh Sheth

**Affiliations:** 1 FRIGE’s Institute of Human Genetics, FRIGE House, Ahmedabad-380015, Gujarat, India

## Background

Molecular study is considered to be the gold standard for single gene disorders. For Lysosomal storage disorders (LSDs), plethora of literature report indicates the use of lysosomal enzyme activity in uncultured chorionic villus sample (CV), cultured chorionic villus (CT) or cultured amniotic fluid cells (AF) as a reliable tool for prenatal diagnosis (PD) followed by mutation study as a confirmation where mutation is known and/or unequivocal enzyme activity is observed. Very few studies are available from India with anecdotal reports using either molecular methods or enzyme study in PD of LSDs. Nonetheless considering the reported prevalence of 1:5,000-7,000 live birth and limited availability of therapeutic option PD remains the only preventable cure for storage disorders.

## Aim

To establish prenatal diagnosis of LSDs and demonstrate the reliable use of lysosomal enzyme study for prenatal diagnosis.

## Material and method

One hundred twenty pregnancies with confirmed index case of LSD were selected for lysosomal enzymes study from CV, CT and AF. Written consent was obtained from guardian of the study subjects.

## Results

Of 120 pregnancies, 57 (47.5%) were normal, 8 (6.66%) had an intermediate enzyme values and rest 55 (45.8%) were found to be affected with specific LSD. From the affected fetuses, 11 (9.1%) were affected with MPS-I, 2 (1.7%) with MPS-II, 1 (0.8%) with MPS-IIIA, 1 (0.8%) with MPS-IIIB, 4 (3.4%) with MPS-IVA, 2 (1.7%) with MPS-VI, 7 (5.8%) with GM-1 gangliosidosis, 1 (0.8%) with Gaucher, 3 (2.5%) with Tay-sachs, 2 (1.7%) with Sandhoff, 1 (0.8%) with NPD-A/B, 1 (0.8%) with MLD, 9 (7.5%) with Krabbe, 4 (3.4%) with Pompe, 2 (1.7%) with Batten and 4 (3.4%) with Mucolipidosis-II/III. All affected fetuses have shown very low enzyme activity (~0-15% of normal mean). We have also identified 8 (6.66%) pregnancies with intermediate enzyme activity (~50% of mean) this includes 3 (2.5%) fetuses were carrier with Sandhoff, 2 (1.7%) with Mucolipidosis-II/III, each one (0.8% for each) with MLD, NPD-A/B and Tay-sachs disease. Except one case of MPS-IVA who was found to have carrier (~30% enzyme activity) during prenatal diagnosis was found to be affected with MPS-IVA after delivery.

## Conclusions

Prenatal diagnosis of LSDs can be made with equal sensitivity and specificity of the molecular study using CV, CT and AF. Nonetheless carrier identification needs to be confirmed by molecular analysis.

